# NLRP3 inflammasome-dependent and -independent interleukin-1β release by macrophages exposed to wear and corrosion products from CoCrMo implants

**DOI:** 10.1371/journal.pone.0334912

**Published:** 2025-11-18

**Authors:** Nasteho Abdoulkader, Jennifer Archibald, Morgane Lignereux, Eric A. Lehoux, Isabelle Catelas

**Affiliations:** 1 Department of Mechanical Engineering, Faculty of Engineering, University of Ottawa, Ottawa, Ontario, Canada; 2 Department of Surgery, Faculty of Medicine, University of Ottawa, Ottawa, Ontario, Canada; 3 Department of Biochemistry, Microbiology, and Immunology, Faculty of Medicine, University of Ottawa, Ottawa, Ontario, Canada; University of the Pacific, UNITED STATES OF AMERICA

## Abstract

Wear particles and metal ions released from cobalt–chromium–molybdenum (CoCrMo) implants can trigger adverse local tissue reactions (ALTR) that can lead to implant failure. Identifying mechanisms involved in ALTR, particularly those underlying the initial inflammatory response elicited by wear particles (Cr_2_O_3_ and CoCrMo) and metal ions (Co^2+^ and Cr^3+^) is therefore critical. The macrophage pro-inflammatory response to CoCrMo particles, Co^2+^, and Cr^3+^ includes interleukin-1β (IL-1β) release, a process putatively linked to the NLPR3 inflammasome. However, the effects of Cr_2_O_3_ particles remain largely unknown. The objectives of this study were to determine whether IL-1β release by macrophages exposed to Cr_2_O_3_ particles (60 nm), CoCrMo particles (3.4 μm), Co^2+^, or Cr^3+^ is dependent on NLRP3 and caspase-1, whether NLRP3-dependent release is mediated by reactive oxygen species (ROS) and/or cathepsin B, and whether caspase-8 is involved when the release is NLRP3 independent. Bone marrow-derived macrophages (BMDM) from wild-type (*wt*), NLRP3-deficient (*Nlrp3*^*-/-*^), and caspase-1-deficient (*Casp1*^*-/-*^) mice were exposed to the particles or metal ions following priming with lipopolysaccharide. IL-1β release induced by Cr_2_O_3_ particles and Cr^3+^ was shown to be both NLRP3 and caspase-1-dependent. In contrast, IL-1β release induced by CoCrMo particles and Co^2+^ occurred independently of NLRP3, being caspase-1-independent in response to CoCrMo particles and partially caspase-1-dependent in response to Co^2+^. Further analysis suggested that NLRP3 inflammasome activation by Cr_2_O_3_ particles was cathepsin B dependent and mediated by lysosomal destabilization, whereas activation by Cr^3+^ was ROS-mediated. NLRP3-independent IL-1β release induced by CoCrMo particles or Co^2+^ was caspase-8 dependent. Collectively, these findings highlight the diversity and specificity of the mechanisms by which different CoCrMo implant wear particles and metal ions can induce IL-1β release in macrophages. Moreover, they suggest that targeting NLRP3, caspase-1, and/or caspase-8 could help mitigate the IL-1β-mediated component of the inflammatory response triggered by wear particles and metal ions from CoCrMo implants.

## Introduction

Cobalt–chromium–molybdenum (CoCrMo) alloys are widely used in medical devices [1], including joint implants. Wear and corrosion of these implants represent a major clinical concern [1], as they have been associated with adverse local tissue reactions (ALTR) [2–4], which can ultimately lead to implant failure [5,6]. ALTR encompass delayed-type IV hypersensitivity-like reactions [7] and pseudotumors [8,9], both frequently accompanied by tissue necrosis [2,10]. Therefore, identifying mechanisms involved in ALTR, particularly those underlying the initial inflammatory response elicited by wear particles and metal ions, may enable the development of non-invasive therapies that improve the lifespan of joint replacements.

Wear of articulating surfaces made of CoCrMo has been reported to primarily generate round to oval-shaped chromium oxide (Cr_2_O_3_) nanoparticles (<100 nm) along with a smaller proportion of larger CoCrMo particles [11,12]. In addition to wear, corrosion – particularly at modular interfaces – can lead to the release of metal ions, including Co^2+^ and Cr^3+^ [13–15]. Macrophages are considered key mediators of the immune response to implant-derived wear and corrosion products [16,17]. Upon classical activation, they release pro-inflammatory cytokines such as interleukin-1β (IL-1β) and tumor necrosis factor alpha (TNF-α), which contribute to the induction and maintenance of a pro-inflammatory response. It is well established that macrophage response to metallic wear particles (e.g., CoCrMo and commercially pure [cp] Ti) [18–20] as well as to metal ions (e.g., Co^2+^, Cr^3+^, and Ni^2+^) involve activation of Toll-like receptors (TLRs) [21,22]. More specifically, an upregulation of TLR-1, -4, and -6 in response to CoCrMo particles or a cocktail of metal ions, generated by oxidation of the alloy, was recently reported [23]. In addition, exposure of macrophages to CoCrMo particles, cpTi particles, or metal ions such as Co^2+^ and Cr^3+^ has been reported to induce activation of the NLRP3 inflammasome resulting in the release of IL-1β [24–26]. Exposure of macrophages to Cr^3+^, unlike exposure to Co^2+^ or Ni^2+^, has also been shown to induce caspase-1 cleavage in the absence – but not in the presence – of an antioxidant, suggesting that Cr^3+^ activates the NLRP3 inflammasome through reactive oxygen species (ROS)-dependent mechanisms [27]. However, to the best of our knowledge, the potential involvement of Cr_2_O_3_ particles in the activation of the NLRP3 inflammasome and release of IL-1β by macrophages remains unexplored.

The NLRP3 inflammasome consists of a sensor protein (NLR family, pyrin domain-containing 3 [NLRP3]), an adaptor protein (apoptosis-associated speck-like protein containing a caspase recruitment domain [ASC]), and an effector protein (caspase-1). Canonical activation of this tripartite complex requires two distinct signals. The first, known as priming, is initiated when TLRs recognize and bind danger-associated molecular patterns (DAMPs) or pathogen-associated molecular patterns (PAMPs) such as bacterial lipopolysaccharide (LPS) – a well-characterized and potent TLR4 agonist derived from Gram-negative bacteria [28]. The second signal, often referred to as a danger signal, is detected by cytosolic NLRP3 and leads to recruitment of the adaptor protein and the zymogen protease, pro-caspase-1 [28,29]. Cellular events capable of serving as this second signal include: ion fluxes (K^+^ efflux, Ca^2+^ influx), ROS generation, and lysosomal destabilization [30]. Following dual-signal activation, the NLRP3 inflammasome assembles, inducing auto-catalytic cleavage and activation of caspase-1, which in turn proteolytically processes its substrates, including pro-IL-1β, into their mature biologically active forms [31].

The mechanisms by which cytosolic and/or mitochondrial ROS induce assembly of the NLRP3 inflammasome complex remain ill-defined [30]. In contrast, it is well-established that lysosomal destabilization can be triggered by particulates, including silica crystals, aluminum salts, and other agents that compromise lysosomal membrane integrity [32,33]. Membrane destabilization can cause the release of lysosomal cathepsins, including cathepsin B, into the cytosol. This release can be functionally important, as cathepsin B has been shown to interact with NLRP3 at the endoplasmic reticulum and its absence has been shown to inhibit NLRP3 inflammasome complex formation [34]. Interestingly, IL-1β release has also been reported to occur without involvement of the NLRP3 inflammasome. For example, while cathepsins [35] have been shown to promote pro-IL-1β synthesis, granzyme A [36] and caspase-8 [37] have been reported to proteolytically cleave pro-IL-1β into mature biologically active forms.

The mechanisms by which CoCrMo implant wear particles and metal ions induce NLRP3 inflammasome-dependent or -independent release of IL-1β by macrophages remain largely unexplored. Therefore, the objectives of the present study were to determine whether IL-1β release by bone marrow-derived macrophages (BMDM) exposed to Cr_2_O_3_ particles (60 nm), CoCrMo particles (3.4 μm), Co^2+^, or Cr^3+^ is dependent on NLRP3 and caspase-1, whether NLRP3-dependent release is mediated by ROS and/or cathepsin B, and whether caspase-8 is involved when the release is NLRP3 independent.

## Materials and methods

### Particles and metal ions

Suspensions of commercially available spherical Cr_2_O_3_ nanoparticles (60-nm average diameter, *as per* the manufacturer; 99% purity; MilliporeSigma, St. Louis, MO, catalog no. 634239) and spherical CoCrMo alloy microparticles (3.4-μm average diameter; d_10_ = 2.4 μm, d_50_ = 3.4 μm, d_90_ = 5.3 μm; composition: Co 59.2%, Cr 30%, Mo 7%, *as per* the manufacturer; Praxair Surface Technologies, Indianapolis, IN, catalog no. ZCO538-J2010) were prepared as previously described [38]. The mass of particles required to achieve the desired concentrations was calculated based on the density of chromium oxide (5.21 g/cm^3^) and CoCrMo alloy (4.86 g/cm^3^) as well as the spherical geometry of the particles. Stock solutions of metal ions were prepared from their corresponding chloride salts, as previously described [27]. Endotoxin levels were assessed using a chromogenic *Limulus* amebocyte lysate (LAL) assay kit (GenScript, Piscataway, NJ). Briefly, the Cr_2_O_3_ and CoCrMo particles (prepared as described above, but in siliconized microcentrifuge tubes; Fisher Scientific, Waltham, MA) were resuspended – at the highest concentrations used in the experiments – in pre-warmed (37°C) endotoxin-free cell culture-grade water (MilliporeSigma) and incubated for 1 h at room temperature with mixing by inversion every 10 min. The suspensions were then centrifuged (21,000 × *g* for 15 min) and the supernatants were assayed *as per* the manufacturer’s instructions. Control samples spiked with 0.05 endotoxin unit (EU) of endotoxin, were used to assess and correct for potential interference from the particles. The supernatants, and thus presumably the particle suspensions, contained ≤ 0.01 EU/mL.

Particle-induced cytotoxicity in BMDM was assessed across a range of concentrations using dye-exclusion hemocytometry (S8 Fig). Corresponding data for the metal ions were previously published by our group [27].

### Antioxidant and inhibitors

Stock solutions of N-acetyl-L-cysteine (NAC, 180 mM), an antioxidant, were freshly prepared by dissolving NAC (MilliporeSigma) into pre-warmed (37°C) phosphate-buffered saline (PBS) without Ca^2+^ and Mg^2+^ (Wisent, St-Jean Baptiste, QC). The solutions were sterile-filtered using a syringe filter equipped with a 0.2-μm pore-size cellulose acetate membrane (VWR, Radnor, PA). Stock solutions of CA-074 methyl ester (CA-074Me, 25 mM) were prepared by dissolving CA-074Me (Cayman Chemical, Ann Arbor, MI) into anhydrous dimethyl sulfoxide (DMSO; MilliporeSigma) pre-sparged with argon (Linde Canada, Mississauga, ON). The solutions were sterile-filtered using a syringe filter equipped with a 0.2-μm pore-size nylon membrane (Cytiva, Marlborough, MA). Aliquots, purged with argon, were frozen and stored in airtight polypropylene vials (Simport, Beloeil, QC) at −20°C until use. Finally, stock solutions (5 mM) of Z-Ile-Glu(O-Me)-Thr-Asp(O-Me) fluoromethyl ketone (Z-IETD-FMK), an inhibitor of caspase-8, were prepared by dissolving Z-IETD-FMK (InvivoGen, San Diego, CA) in anhydrous DMSO. The solutions were sterile-filtered using a syringe filter equipped with a 0.2-μm pore-size nylon membrane. Aliquots were frozen and stored at −20°C until use.

### Animals

**Fig 1:**
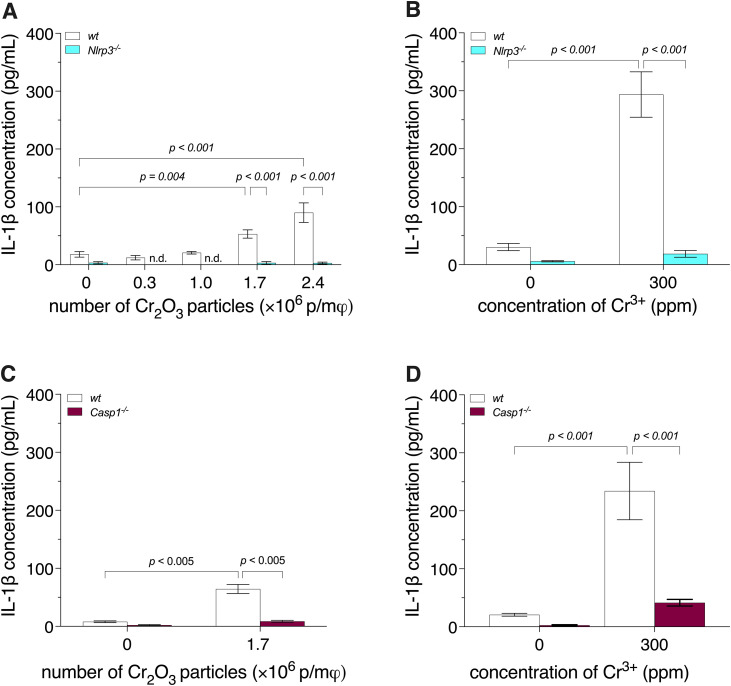
IL-1β release by BMDM, from wild-type (*wt*), *Nlrp3*^-/-^, and *Casp1*^-/-^ mice, exposed to Cr_2_O_3_ particles or Cr^3+^. Bone marrow-derived macrophages (BMDM) were primed with 500 ng/mL of lipopolysaccharide for 3 h, then exposed to either 60-nm spherical Cr_2_O_3_ particles (**A**, **C**) or Cr^3+^ (**B, D**) for 18 h. Interleukin-1β (IL-1β) release was quantified by enzyme-linked immunosorbent assay (ELISA). Two-way analyses of variance (ANOVA) were performed **(A, B)**. Since an interaction (*p* < 0.001) was detected, Holm-Šídák multiple-comparison post hoc tests were conducted, **(A, B)**. Welch’s t-tests with Benjamini-Hochberg correction were performed **(C, D)**. Data are presented as means ± SEM of 3 (**A**, **B**) or 4 (**C**, **D**) independent experiments. n.d.: not detected. p/mφ:particles per macrophage.

All procedures were approved by the University of Ottawa Animal Care Committee (Protocols ME-2350 and ME-3363). The University of Ottawa Animal Care and Use Program meets the Canadian Council on Animal Care (CCAC) guidelines and is licensed under the Province of Ontario Animals for Research Act. Mice were purchased from The Jackson Laboratory (Bar Harbor, ME), except for the wild-type (*wt*) mice, used in the experiments reported in Figs 1A, 1C, 1D, 4, and 5, which were purchased from Charles River Laboratories (Montreal, QC). The mice (2–5 per cage) were housed at the Animal Care Facility of the University of Ottawa, a specific-pathogen-free (SPF) facility, in individually ventilated cages (Sealsafe Plus GM500; Techniplast, West Chester, PA) containing 6-mm size corncob bedding (Envigo RMS, Indianapolis, IN), cotton fiber-based nesting material (Ancare, Bellmore, NY), and a shreddable refuge hut (Ketchum, Brockville, ON). They were maintained at 22°C with 40% relative humidity under a 12 h-light:12 h-dark photoperiod with *ad libitum* access to food (2018 Teklad Global 18% Protein Rodent Diet; Envigo RMS) and water (purified by reverse osmosis and acidified to pH 2.5–3.0 with hydrochloric acid). NLRP3-deficient (*Nlrp3*^*-/-*^), caspase-1-deficient (*Casp1*^*-/-*^), and *wt* female C57BL/6 mice, 6–17 weeks old (body mass: 20 ± 0.5 g), 4–7 weeks old (body mass 20 ± 0.1 g), and 5–18 weeks old (body mass: 20 ± 1.4 g), respectively, were euthanized between 8h00 and 15h00 by CO_2_ gas inhalation, followed by cervical dislocation after loss of consciousness – in compliance with CCAC guidelines requiring a secondary physical method to ensure death in small rodents. To minimize distress, euthanasia was performed in the animals’ home cage, using a gradual CO_2_ fill rate of 20–30% of the chamber volume per min, in accordance with CCAC guidelines and institutional animal care protocols. Euthanized mice were soaked with 70% (v/v) ethanol immediately prior to dissection.

**Fig 2:**
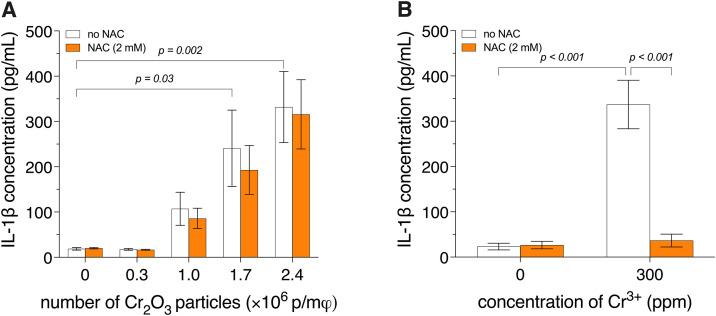
IL-1β release by BMDM exposed to Cr_2_O_3_ particles or Cr^3^^+^ with or without NAC. Bone marrow-derived macrophages (BMDM) from wild-type mice were primed with 500 ng/mL of lipopolysaccharide for 3 h, then exposed to 60-nm spherical Cr_2_O_3_ particles (**A**) or Cr^3+^ (**B**) for 18 h, in the presence or absence of N-acetyl-L-cysteine (NAC), an antioxidant. Interleukin-1β (IL-1β) release was quantified by enzyme-linked immunosorbent assay (ELISA). Two-way analyses of variance (ANOVA) were performed. Holm-Šídák pairwise post hoc tests were conducted for panel **(A)**. For panel **(B)**, the Holm-Šídák multiple-comparison post hoc test was used following the detection of an interaction (*p* < 0.001). Data are presented as means ± SEM of 3 independent experiments. p/mφ: particles per macrophage.

**Fig 3:**
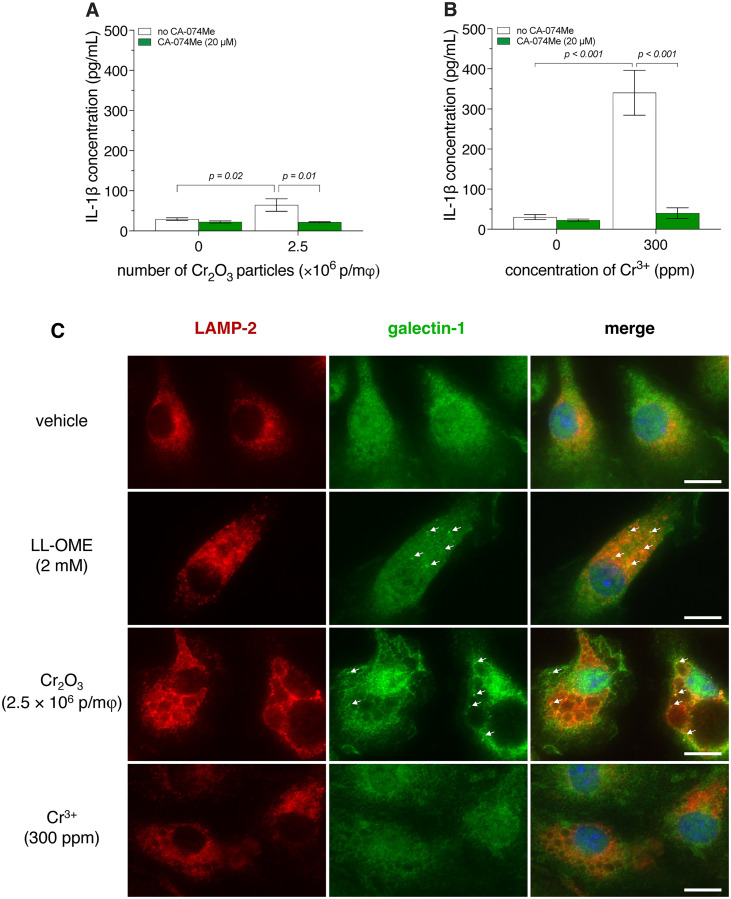
IL-1β release and galectin-1 puncta formation in BMDM exposed to Cr_2_O_3_ particles or Cr^3^^+^ with or without CA-074Me. Bone marrow-derived macrophages (BMDM) from wild-type mice were primed with 500 ng/mL of lipopolysaccharide for 3 h, then exposed to 60-nm spherical Cr_2_O_3_ particles or Cr^3+^ for 18 h, in the presence or absence of CA-074 methyl ester (CA-074Me), a cathepsin B inhibitor. Interleukin-1β (IL-1β) release (**A**, **B**) was quantified by enzyme-linked immunosorbent assay (ELISA), and galectin-1 puncta formation (**C**) was visualized using fluorescently labeled anti-lysosome-associated membrane protein 2 (LAMP-2, red) and anti-galectin-1 (green) antibodies. Nuclei were stained with 4,6-diamidino-2-phenylindole dihydrochloride (DAPI, blue). L-leucyl-L-leucine methyl ester (LL-OMe) was used as a positive control for galectin-1 puncta formation. Note that image brightness and contrast were digitally optimized for visual clarity. Therefore, signal intensity is not directly comparable between images. Arrows point to galectin-1 puncta. Two-way analyses of variance (ANOVA) were performed. Holm-Šídák pairwise post hoc tests were conducted for panel **(A)**. For panel **(B)**, the Holm-Šídák multiple-comparison post hoc test was used following the detection of an interaction (*p* < 0.001). Data are presented as means ± SEM of 3 independent experiments. p/mφ: particles per macrophage. Scale bar: 10 μm.

### Bone marrow-derived macrophages

**Fig 4:**
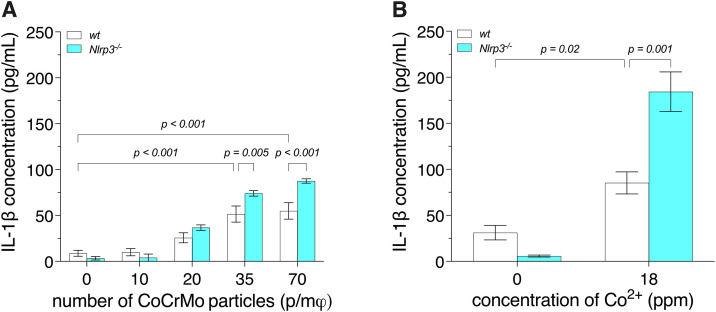
IL-1β release by BMDM, from wild-type (*wt*) and *Nlrp3*^-/-^ mice, exposed to CoCrMo particles or Co^2^^+^. Bone marrow-derived macrophages (BMDM) were primed with 500 ng/mL of lipopolysaccharide for 3 h, then exposed to 3.4-μm spherical CoCrMo particles (**A**) or Co^2+^ (**B**) for 18 h. Interleukin-1β (IL-1β) release was quantified by enzyme-linked immunosorbent assay (ELISA). Two-way analyses of variance (ANOVA) were performed. Since an interaction (*p* = 0.003 **[A]** and *p *= 0.001 **[B]**) was detected, Holm-Šídák multiple-comparison post hoc tests were conducted **(A, B)**. Data are presented as means ± SEM of 3 independent experiments. p/mφ: particles per macrophage.

**Fig 5:**
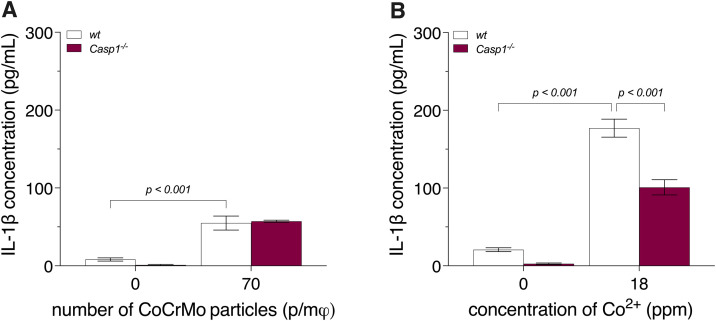
IL-1β release by BMDM, from wild-type (*wt*) and *Casp1*^-/-^ mice, exposed to CoCrMo particles or Co^2^^+^. Bone marrow-derived macrophages (BMDM) were primed with 500 ng/mL of lipopolysaccharide for 3 h, then exposed to 3.4-μm spherical CoCrMo particles (**A**) or Co^2+^ (**B**) for 18 h. Interleukin-1β (IL-1β) release was quantified by enzyme-linked immunosorbent assay (ELISA). Two-way analyses of variance (ANOVA) were performed. Holm-Šídák pairwise post hoc tests were conducted for panel **(A)**. For panel **(B)**, the Holm-Šídák multiple-comparison post hoc test was used following the detection of an interaction (*p* = 0.003). Data are presented as means ± SEM of 3 independent experiments. p/mφ: particles per macrophage.

**Fig 6:**
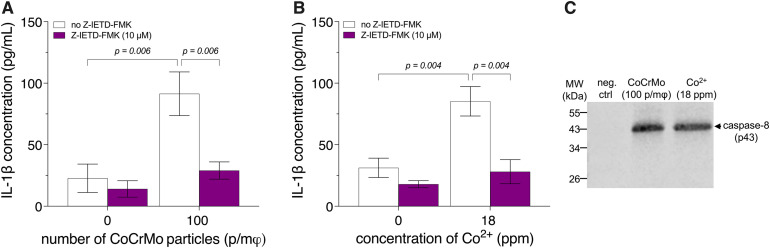
IL-1β release, with and without a caspase 8 inhibitor, and caspase-8 cleavage in BMDM exposed to CoCrMo particles or Co^2^^+^. Bone marrow-derived macrophages (BMDM) from wild-type mice were primed with 500 ng/mL of lipopolysaccharide for 3 h, then exposed to 3.4-μm spherical CoCrMo particles or Co^2+^ for 18 h in the presence or absence of a caspase-8 inhibitor (Z-IETD-FMK). Interleukin-1β (IL-1β) release (**A**, **B**) was quantified by enzyme-linked immunosorbent assay (ELISA). Cell lysates were analyzed for caspase-8 processing (**C**) by immunoblotting. A representative immunoblot is shown. All (full-size) immunoblot replicates (including total protein staining) are shown in S7 Fig. Two-way analyses of variance (ANOVA) were performed. Since an interaction (*p* = 0.05 **[A]** and *p* = 0.04 **[B]**) was detected, Holm-Šídák multiple-comparison post hoc tests were conducted **(A, B)**. Data are presented as means ± SEM of 3 independent experiments. neg. ctrl: negative control (vehicle). p/mφ: particles per macrophage.

Bone marrow cells were harvested from tibiae and femora isolated from euthanized mice and prepared as previously described [27], except that Dulbecco’s modified Eagle medium (DMEM)-based complete growth medium (CGM) was used. The CGM consisted of DMEM (Wisent) supplemented with 8% (v/v) heat-inactivated fetal bovine serum (FBS; < 0.15 EU/mL endotoxin; NorthBio, Toronto, ON) and 100 U/mL each of penicillin and streptomycin (Cytiva). Cell counts were performed using dye-exclusion hemocytometry with 0.04% (w/v, final) trypan blue (MilliporeSigma) and an Improved Neubauer hemocytometer (Hausser Scientific, Horsham, PA). Cells were then seeded at ca. 230,000 cells/cm^2^ in polystyrene Petri dishes (Greiner Bio-One, Monroe, NC) coated with recombinant macrophage colony stimulating factor (M-CSF, 0.85 ug/cm^2^; R&D systems, Minneapolis, MN), as described by Sadh *et al.* [39]. The seeded cells were incubated for 6 days in CGM supplemented with β-mercaptoethanol (55 μM final; ThermoFisher Scientific, Rockford, IL), under cell culture conditions (37°C, humidified atmosphere of 95% air and 5% CO_2_). At the end of the incubation, non-adherent cells were removed by rinsing with CGM, and BMDM were detached either by pipetting using a 10-mL Class A volumetric glass pipette (Sibata Scientific Technology, Soka, Japan) or using a 2-cm-blade polyethylene cell lifter (Fisher Scientific). BMDM were collected by centrifugation (300 × g for 10 min) and resuspended at 1.0 × 10^6^ cells/mL in pre-warmed (37°C) CGM supplemented (Figs 2, 3, 6, S2 Fig, S3 Fig, and S6 Fig) or not (Figs 1, 4, 5, and S8 Fig) with M-CSF (5 ng/mL final). Supplementation was implemented following the observation that it enhanced cell viability during the recovery period. Flow cytometric immunophenotyping (S1 Materials and methods) indicated that ca. 95% of the cells in the BMDM preparations were macrophages (S2 Fig).

### Interleukin-1β and tumor necrosis factor-α release

BMDM were seeded at 1.6 × 10^5^ cells/cm^2^ in 24-well tissue culture-treated polystyrene plates (Greiner Bio-One) and incubated overnight in CGM, supplemented or not with M-CSF (5 ng/mL final), to allow recovery from harvesting. The following day, BMDM were primed in CGM freshly supplemented with LPS from *E. coli* O55:B5 (500 ng/mL; MilliporeSigma) for 3 h under cell culture conditions, as previously described [27]. The effects of priming with different LPS concentrations on IL-1β release are shown in S3 Fig. Primed BMDM from *wt* mice were pre-incubated for 1 h under cell culture conditions in fresh CGM, unsupplemented (negative control), or supplemented with 2 mM NAC, 20 μM CA-074Me, or 10 μM Z-IETD-FMK. The cells were then exposed to Cr_2_O_3_ particles (0–2.4 × 10^6^ particles/macrophage [p/mφ]), CoCrMo particles (0–70 p/mφ), Cr^3+^ (300 ppm), Co^2+^ (18 ppm), or CGM (vehicle) for 18 h. Where appropriate (Figs 1, 4, and 5), nigericin (15 μM; Cayman Chemical) – a potassium ionophore and a potent NLRP3 inflammasome inducer – was used as a positive control. The incubation period (18 h) with the particles, metal ions, or nigericin was selected as a compromise that allows sufficient time for stimulation while minimizing cell death. At the end of the incubation, culture supernatants were collected, centrifuged (21,000 × *g* for 10 min), flash-frozen in liquid nitrogen, and stored at −80°C for subsequent cytokine analysis. IL-1β and TNF-α were analyzed using enzyme-linked immunosorbent assays (ELISA; ThermoFisher Scientific, catalog nos. 88-7013A-77 and 88-7324-88; nominal detection limit: 8 pg/mL and 15.6 pg/mL, respectively), *as per* the manufacturer’s instructions. Interference from CGM, Co^2+^, or Cr^3+^ with IL-1β quantification by ELISA was ca. 6% (negative interference), 5% (positive interference), and 14% (negative interference), respectively, as shown in S4 Fig. No correction was applied to the data since the magnitude of the interference was relatively small, and the primary aim of the present study was to identify mechanisms underlying IL-1β release rather than to precisely quantify its absolute levels, which can vary depending on experimental conditions.

### Galectin puncta formation

Lysosomal membrane permeabilization was assessed using the galectin puncta formation assay [40]. Briefly, BMDM from *wt* mice were seeded, as described above, in 18-well chamber slides (no. 1.5 tissue-culture treated proprietary polymer coverslip; Ibidi, Gräfelfing, Germany), and incubated overnight with 5 ng/mL M-CSF. The cells were then primed as described above and exposed to Cr_2_O_3_ particles (2.5 × 10^6^ p/mφ), Cr^3+^ (300 ppm), or CGM (vehicle) for 6 h, or to L-leucyl-L-leucine methyl ester (LL-OMe; 2 mM; Cayman Chemical) for 2 h as a positive control for lysosomal membrane permeabilization, all under cell culture conditions. At the end of the exposure, attached cells were fixed in buffered paraformaldehyde solution (4% w/v in PBS; ThermoFisher Scientific) for 10 min, then washed with PBS without Ca^2+^ and Mg^2+^ (Wisent). The fixed cells were incubated for 20 min in blocking buffer (1% w/v bovine serum albumin [MilliporeSigma], 0.3% v/v Triton X-100 [MilliporeSigma], and 5% v/v FBS, in PBS without Ca^2+^ and Mg^2+^), followed by another wash with PBS without Ca^2+^ and Mg^2+^. They were then co-immunostained by overnight incubation at 4°C with recombinant rabbit anti-galectin-1 monoclonal antibody [EPR3205] (1:1000 dilution in blocking buffer; Abcam, Cambridge, UK; catalog no. ab108389) and rat anti-lysosome-associated-membrane protein 2 (LAMP-2) monoclonal antibody [GL2A7] (1:1000 dilution in blocking buffer; Abcam, catalog no. ab13524). The cells were then washed three times with staining buffer (0.25% w/v bovine serum albumin [BSA; Millipore Sigma] and 0.1% v/v Triton X-100 in PBS without Ca^2+^ and Mg^2+^) and co-incubated for 1 h with polyclonal secondary antibodies: donkey anti-rabbit IgG (H + L) conjugated to Alexa Fluor 488 (1:1000 dilution in staining buffer; ThermoFisher Scientific, RRID: AB 2535792) and donkey anti-rat IgG (H + L) conjugated to Alexa Fluor 594 (1:1000 dilution in staining buffer; ThermoFisher Scientific, RRID: AB 2535795). Nuclei were stained with 4,6-diamidino-2-phenylindole dihydrochloride (DAPI; 0.1% w/v in PBS without Ca^2+^ and Mg^2+^; MilliporeSigma) for 10 min. Galectin puncta formation was visualized using an inverted epifluorescence widefield microscope (Axio Observer Z1; Zeiss, Oberkochen, Germany). The stand was equipped with a light-emitting diode (LED) light source (X-Cite Xylis; Excelitas Technologies; Pittsburgh, PA) and a monochrome charge-coupled device (CCD) camera (Axiocam MRm, Zeiss). Image acquisition parameters were configured using ZEN (Blue Edition) v2.3 software (Zeiss), as detailed below. Images were acquired using a Plan-Apochromat 63 × oil-immersion differential interference contrast objective with a 1.4 numerical aperture (Zeiss). Fluorescence imaging was performed using the following filter cubes: blue (G 365 nm LED peak excitation, *farb teiler* [FT] 395 nm dichromatic beamsplitter, long-pass [LP] 420 nm emission) for DAPI, green (band-pass [BP] 470/40 nm excitation, FT 495 nm, BP 525/50 nm emission) for Alexa Fluor 488, and orange (BP 546/12 nm [high efficiency; HE] excitation, FT 560 nm [HE], BP 607/80 nm [HE] emission) for Alexa Fluor 594. The light source power output and the exposure time were 8% and 100 ms for DAPI, 20% and 250 ms for Alexa Fluor 488, and 20% and 300 ms, for Alexa Fluor 594, respectively. z-stacking was performed with a step size of 120 nm.

### Caspase-8 processing

Caspase-8 processing was analyzed by immunoblotting. BMDM from *wt* mice were seeded, as above, in 6-well tissue culture-treated polystyrene plates (Greiner Bio-One), incubated overnight in CGM supplemented with 5 ng/mL M-CSF, primed as described above, then incubated for 18 h with either CoCrMo particles (100 p/mφ), Co^2+^ (18 ppm), or CGM (vehicle). At the end of the incubation, the plates were placed on ice and the cells washed twice with ice-cold PBS without Ca^2+^ and Mg^2+^. The cells were then detached in ice-cold PBS without Ca^2+^ and Mg^2+^, supplemented with an ethylenediaminetetraacetic acid (EDTA)-free protease inhibitor cocktail (cOmplete; MilliporeSigma), *as per* the manufacturer’s instructions, using a cell lifter. Cells were collected by centrifugation (10,000 × *g* for 5 min at 4°C), and resuspended in 50 µL of ice-cold lysis buffer composed of 125 mM Tris-HCl (pH 6.8; Fisher Scientific), 2% (w/v) sodium dodecyl sulfate (SDS; Fisher Scientific), 10% (v/v) glycerol (MilliporeSigma), and 100 mM DL-1,4-dithiothreitol (VWR). Cells lysis was performed by sonication on ice for 15 s using an ultrasonic processor (Model 120 Sonic Dismembrator; Fisher Scientific) set to 20% amplitude, fitted with a 1/8-inch probe microtip (Model CL-18; Fisher Scientific). The lysates were centrifuged (20,000 × *g* for 15 min at 4°C) and the supernatants were collected and stored at −20°C until use. Aliquots containing 40 μg of total protein, as determined using a colorimetric assay with BSA as the protein standard (Pierce 660 nm Protein Assay with Ionic Detergent Compatibility Reagent; ThermoFisher Scientific), were mixed with 4 × protein loading buffer (LI-COR, Lincoln, NE), *as per* the manufacturer’s instructions. Samples were then analyzed by SDS-polyacrylamide gel electrophoresis (SDS-PAGE) using precast mini-format Tris-glycine gradient gels (8–16%; Bio-Rad; Hercules, CA). A prestained protein ladder (visible and near-infrared [NIR]; LI-COR) served as molecular weight marker. Following electrophoresis, proteins were electrotransferred onto a 0.45-μm pore size fluorescent-grade polyvinylidene fluoride membrane (Immobilon-FL; MilliporeSigma). The membrane was then reversibly stained for total protein with acid blue (REVERT Total Protein Stain; LI-COR), *as per* the manufacturer’s instructions, and imaged at 700 nm using a NIR fluorescence imaging system (Odyssey Fc; LI-COR). The stained membrane was incubated in commercial blocking buffer (Intercept [PBS] Blocking Buffer; LI-COR) for 1 h at room temperature. Immunodetection was performed using a rabbit anti-mouse cleaved caspase-8 antibody (Cell Signaling Technology, catalog no. 9429) diluted 1:1000 in commercial antibody diluent (Intercept T20 [PBS] Antibody Diluent) supplemented with an additional 0.1% (v/v) polysorbate 20 (MilliporeSigma), with overnight incubation at 4°C. The secondary antibody used was a goat anti-rabbit IgG (H + L) fluorescently labeled with 800CW (LI-COR, catalog no. 926–32211), diluted 1:10,000 in commercial antibody diluent (Intercept T20 [PBS] Antibody Diluent) supplemented with 0.01% (w/v) SDS and polysorbate 20 at a final concentration of 0.2% (v/v), and incubated 1 h with the membrane at room temperature. The membrane was washed in PBS without Ca^2+^ and Mg^2+^, air-dried in the dark, and then imaged at 800 nm using the above LI-COR NIR fluorescence imaging system.

### Statistical analysis

Unless otherwise specified, statistical analysis was performed using GraphPad Prism v9.4.1 for macOS. Data were considered to meet the assumptions of normality, and homoscedasticity was assessed using Levene’s test, implemented in R v2025.1.513 [41]. When the assumption of homogeneity was violated, the Welch *t*-test was performed, and the resulting *p* values were adjusted using the Benjamini-Hochberg procedure to control the false discovery rate (FDR) at 0.05. Otherwise, two-way analyses of variance (ANOVA) were performed, followed by the Holm-Šídák test: pairwise tests to analyze main effects, or multiple comparison tests to assess simple main effects when an interaction was found. *p* < 0.05 was considered significant. Significant differences are reported exclusively for the following three types of comparisons: 1) Particle/ion exposure – BMDM (from either *wt* or knockout mice) exposed to a specific particle or metal ion concentration were compared to the negative control (cells unexposed to exogenous particles or metal ions), in the absence of an inhibitor or antioxidant; 2) Genetic manipulation – BMDM from *wt* mice were compared to those from knockout mice, both exposed to the same particle or metal ion concentration; and 3) Inhibitor/antioxidant treatment – BMDM exposed to a specific particle or metal ion concentration were compared in the presence or absence of an inhibitor or antioxidant. Effect sizes, computed manually in Microsoft Excel, are presented in S5 Table as Cohen’s *d* with 95% confidence intervals (CI). Unless otherwise specified, data are presented as mean ± SEM of at least 3 independent experiments, each performed with 3 replicate samples per condition.

## Results

### NLRP3 and caspase-1 dependence of Cr_2_O_3_ particle- and Cr^3+^-induced IL-1β release

To determine the NLRP3 and/or caspase-1 dependence of IL-1β release by macrophages exposed to Cr_2_O_3_ nanoparticles or Cr^3+^, we analyzed IL-1β release by BMDM, from *wt, Nlrp3*^*-/-*^, *or Casp1*^*-/-*^ mice, exposed to 60-nm spherical Cr_2_O_3_ particles or Cr^3+^ (Fig 1). Exposure of BMDM from *wt* mice to the particles induced a particle concentration-dependent increase in IL-1β release, reaching ca. 400% with 2.4 × 10^6^ p/mφ (*p* < 0.001), relative to the negative control (BMDM unexposed to Cr_2_O_3_ particles; Fig 1A). Similarly, exposure to Cr^3+^ (300 ppm) induced an increase in IL-1β release of ca. 870% (*p* < 0.001), relative to the negative control (BMDM unexposed to Cr^3+^; Fig 1B). In contrast, IL-1β release by BMDM from *Nlrp3*^*-/-*^ mice was less than 7% of that observed by BMDM from *wt* mice when the cells were exposed to Cr_2_O_3_ particles (e.g., 3 ± 1% with 2.4 × 10^6^ p/mφ; *p* < 0.001) or Cr^3+^ (300 ppm; 6 ± 2%; *p* < 0.001). Similarly, IL-1β release by BMDM from *Casp1*^*-/-*^ mice was ca. 17% (*p* = 0.005) of that by BMDM from *wt* mice when the cells were exposed to 1.7 × 10^6^ Cr_2_O_3_ p/mφ (Fig 1C) and ca. 13% (*p* < 0.001) of that by BMDM from *wt* mice when exposed to Cr^3+^ (300 ppm; Fig 1D). Finally, exposure of BMDM from *wt* mice to the positive control, nigericin (15 μM), induced a large increase in IL-1β release (*p* < 0.001), but had no statistically significant effect on BMDM from *Nlrp3*^*-/-*^ mice (S6 Fig).

### ROS dependence of Cr_2_O_3_ particle- and Cr^3+^-induced NLRP3 inflammasome activation

ROS are established mediators of NLRP3 inflammasome activation. To assess whether IL-1β release by macrophages exposed to Cr_2_O_3_ nanoparticles or Cr^3+^ is ROS dependent, we analyzed the effect of NAC, an antioxidant, on this release. IL-1β release by BMDM, from *wt* mice, exposed to 60-nm Cr_2_O_3_ particles (up to 2.4 × 10^6^ p/mφ) was not statistically significantly affected by NAC (Fig 2A). In sharp contrast, IL-1β release by BMDM, from *wt* mice, exposed to Cr^3+^ (300 ppm) was ca. 90% lower (*p* < 0.001; Fig 2B) in the presence of NAC, i.e., similar to that of the negative control.

### Cathepsin B dependence of Cr_2_O_3_ particle- and Cr^3+^-induced IL-1β release

Cathepsin B is a known mediator of NLRP3 inflammasome activation. To determine whether IL-1β release by macrophages exposed to Cr_2_O_3_ nanoparticles or Cr^3+^ is dependent on cathepsin B activity, we analyzed the effect of CA-074Me, a cathepsin B inhibitor, on this release. In the presence of CA-074Me, IL-1β release by BMDM, from *wt* mice, exposed to 60-nm Cr_2_O_3_ particles (2.5 × 10^6^ p/mφ) or Cr^3+^ (300 ppm) was inhibited by ca. 70% (*p* = 0.01) and 84% (*p* < 0.001), respectively – resulting in levels similar to that of the negative control (Fig 3A and B, respectively). To assess whether the cathepsin B dependence of IL-1β release by macrophages exposed to 60-nm Cr_2_O_3_ particles or Cr^3+^ results from lysosomal leakage of the enzyme, we examined galectin-1 puncta formation – an indicator of lysosomal destabilization – in BMDM from *wt* mice. Upon lysosomal permeabilization, galectin-1, a small cytosolic carbohydrate-binding protein, translocates to the inner surface of the lysosomal membrane. This translocation can be visualized by immunofluorescence as a shift from diffuse to punctate staining [40]. As shown in Fig 3C, galectin-1 puncta formation was observed in BMDM exposed to Cr_2_O_3_ particles (2.5 × 10^6^ p/mφ), but not in those exposed to Cr^3+^ (300 ppm).

### NLRP3 independence of CoCrMo particle- and Co^2+^-induced IL-1β release

To analyze the NLRP3 dependence of IL-1β release by macrophages exposed to CoCrMo microparticles or Co^2+^, we compared the release of IL-1β by BMDM from *wt* and *Nlrp3*^*-/-*^ mice. Exposure of the BMDM from *wt* mice to 3.4-μm CoCrMo particles induced a particle concentration-dependent increase in IL-1β release reaching ca. 525% with 70 p/mφ (*p* < 0.001), relative to the negative control (BMDM unexposed to CoCrMo particles; Fig 4A). Similarly, exposure of BMDM, from *wt* mice, to Co^2+^ (18 ppm) induced an increase in IL-1β release of ca. 175% (*p* = 0.02), relative to the negative control (BMDM unexposed to Co^2+^; Fig 4B). Knocking out *Nlrp3* did not significantly affect IL-1β release by BMDM exposed to CoCrMo particles, and IL-1β release by BMDM from both *wt* and *Nlrp3*^-/-^ mice peaked with 35–70 p/mφ (Fig 4A). Similarly, IL-1β release by BMDM exposed to Co^2+^ was not significantly affected by the absence of NLRP3. Interestingly, knocking out *Nlrp3* increased IL-1β release induced by CoCrMo particles and Co^2+^ by up to 60% with 70 p/mφ (*p* < 0.001; Fig 4A) and 116% with 18 ppm (*p* = 0.001; Fig 4B), respectively.

### Caspase-1 dependence of CoCrMo particle- and Co^2+^-induced IL-1β release

To analyze the caspase-1 dependence of IL-1β release by macrophages exposed to CoCrMo microparticles or Co^2+^, we compared the release of IL-1β by BMDM from *wt* and *Casp1*^*-/-*^ mice. Knocking out *Casp1* did not significantly affect IL-1β release by BMDM exposed to 3.4-μm CoCrMo particles (Fig 5A). In contrast, the absence of caspase-1 resulted in ca. 43% lower IL-1β release by BMDM exposed to Co^2+^ (18 ppm; *p* < 0.001; Fig 5B).

### Caspase-8 dependence of CoCrMo- and Co^2+^-induced IL-1β release

Caspase-8 has been shown to cleave pro-IL-1β into its biologically active form and be able to do so independently of the NLRP3 inflammasome [37]. To assess whether IL-1β release by macrophages exposed to CoCrMo microparticles or Co^2+^ depends on caspase-8, we analyzed the effects of Z-IETD-FMK, a caspase-8 inhibitor, on this release. In the presence of Z-IETD-FMK, IL-1β release by BMDM, from *wt* mice, exposed to 3.4-μm CoCrMo particles (100 p/mφ) or Co^2+^ (18 ppm) was inhibited by ca. 70% (*p* = 0.006 and *p* = 0.004, respectively), resulting in levels similar to that of the negative control (Fig 6A and B, respectively). To confirm that CoCrMo particles and Co^2+^ induced caspase-8 activation, we analyzed caspase-8 cleavage in BMDM from *wt* mice. Immunoblotting revealed that exposure to CoCrMo particles (100 p/mφ) or Co^2+^ (18 ppm) resulted in caspase-8 processing, as evidenced by the detection of the 43 kDa cleaved caspase-8 fragment (Fig 6C).

## Discussion

The present study aimed to elucidate molecular mechanisms by which wear particles (Cr_2_O_3_ and CoCrMo) and metal ions (Co^2+^ and Cr^3+^) from CoCrMo implants lead to IL-1β release by murine BMDM. Distinct activation pathways were observed depending on the stimulus. The release of IL-1β induced by spherical Cr_2_O_3_ nanoparticles or Cr^3+^ was dependent on both NLRP3 and caspase-1, whereas that induced by spherical CoCrMo microparticles or Co^2+^ was NLRP3 independent and either caspase-1 independent (in the case of CoCrMo particles) or partially caspase-1 dependent (for Co^2+^). Further analysis suggested that IL-1β release in response to the Cr_2_O_3_ particles was cathepsin B dependent, implicating cathepsin B-mediated NLRP3 inflammasome activation. In contrast, Cr^3+^-induced IL-1β release was ROS dependent, as shown by antioxidant treatment, suggesting ROS-mediated NLRP3 inflammasome activation. The NLRP3-independent IL-1β release induced by the CoCrMo particles was caspase-8 dependent, based on inhibition and processing evidence, but caspase-1 independent. In contrast, Co^2+^-induced IL-1β release was caspase-8 dependent and partially caspase-1 dependent.

Macrophages have been shown to accumulate in periprosthetic tissues of failed hip implants [17] and play a central role in the immune response to implant wear and corrosion products [42]. Their activation in response to these products leads to the release of pro-inflammatory cytokines, including IL-1β [24,25,27,43]. Murine BMDM, the most common macrophage model in immunological studies [44], were used in the present study for reasons detailed elsewhere [44,45], including their easy availability from genetically modified mouse strains.

The size, shape, concentration, and composition of wear particles are known to influence macrophage response *in vitro* and tissue reactions *in vivo* [12,24,46,47]. In the present study, commercially available spherical Cr_2_O_3_ nanoparticles (60 nm; 0–3.5 × 10^6^ p/mφ) and CoCrMo microparticles (3.4 μm; 0–70 p/mφ) were used. The size and shape of Cr_2_O_3_ particles are consistent with those isolated from periprosthetic tissues of patients with a metal-on-metal hip implant [11,48,49], as well as those generated by hip simulators [11,50]. While the CoCrMo particles are exclusively spherical and larger than those typically found *in vivo*, their size remains within the endocytosable range [51], and their composition is physiologically relevant [11,48,49]. A range of particle concentrations was selected to assess dose-dependent responses, based on the conceptual premise that macrophages *in vitro* require higher concentrations of stimulating agents than they do *in vivo,* where they are exposed to multiple cues. The concentrations of Co^2+^ (18 ppm) and Cr^3+^ (300 ppm) were selected to induce peak IL-1β release, as determined by previous work from our group [27].

IL-1β release has been classically linked with activation of inflammasome complexes, particularly the NLRP3 inflammasome. Sterile inflammation inducers have also frequently been associated with NLRP3 inflammasome activation and subsequent IL-1β release [52]. In the present study, we investigated the dependence of IL-1β release on NLRP3-inflammasome signaling in macrophages exposed to spherical Cr_2_O_3_ (60 nm) or CoCrMo (3.4 μm) particles, or Co^2+^ or Cr^3+^. Exposure of the primed BMDM to CoCrMo particles, Co^2+^, or Cr^3+^ induced IL-1β release, in agreement with previous studies [27,43]. Notably, Cr_2_O_3_ particles, despite their relatively inert ceramic composition, also induced a concentration-dependent IL-1β release. This finding is consistent with earlier reports showing that exposure of THP-1 human monocytes to nearly spherical Cr_2_O_3_ particles (60-nm) [53] or spherical CrOOH particles (15-nm) [54] increased IL-1β gene expression and IL-1β release, respectively. The release of IL-1β induced by Cr_2_O_3_ particles and Cr^3+^ was both NLRP3- and caspase-1-dependent, suggesting NLRP3 inflammasome dependence.

Given the established role of ROS and lysosomal destabilization in NLRP3 inflammasome activation, we investigated whether Cr_2_O_3_ particles and Cr^3+^ can trigger these upstream events. Specifically, the involvement of ROS in Cr_2_O_3_ particle- and Cr^3+^-induced NLRP3-dependent IL-1β release was assessed by measuring the effects of the antioxidant NAC on IL-1β release. Results showed that IL-1β release induced by Cr^3+^, but not by the Cr_2_O_3_ particles, was marginal in the presence of NAC, thereby suggesting a ROS-dependent response. This finding is consistent with a previous study from our group [27], which demonstrated that Cr^3+^-induced IL-1β release by BMDM was marginal in the presence of ascorbic acid, another antioxidant, and that processed caspase-1 (p20 subunit) was detectable only in its absence. Moreover, the marginal IL-1β release induced by Cr^3+^ in the presence of either NAC or ascorbic acid suggests that these effects were primarily due to their antioxidant properties rather than their ability to chelate metal ions [55,56] – as the concentrations were insufficient to extensively chelate 300 ppm of Cr^3+^ – or the role of ascorbic acid as an enzymatic cofactor. Although the mechanisms by which Cr^3+^ induce ROS production remain unclear, proteomics data have shown that Cr^3+^ can directly bind to cellular redox enzymes [57]. Cr^3+^ have also been shown to significantly impair the catalytic activity of catalase, reducing their ability to decompose hydrogen peroxide (H₂O₂) in human erythrocytes [57]. Additionally, Cr^3+^ have been reported to lower glutathione levels in the liver and kidney of goldfish [58]. Together, these findings suggest that Cr^3+^ may increase ROS levels by disrupting key elements of the cellular antioxidant defense system. Interestingly, Salloum *et al.* [59] reported that Cr^3+^ exposure did not significantly increase ROS levels or oxidative stress in RAW 264.7 murine macrophages. This apparent inconsistency with the present findings may be attributed to ROS compartmentalization, the hyperoxic conditions of standard cell culture [60], or methodological distinctions such as the use of primary non-neoplastic cells vs. neoplastic cells.

To investigate the potential role of cathepsin B in NLRP3-dependent IL-1β release induced by the Cr_2_O_3_ particles and Cr^3+^, we assessed the effects of cathepsin B inhibitor CA-074Me on IL-1β release. Results showed that CA-074Me prevented IL-1β release induced by the Cr_2_O_3_ particles as well as by Cr^3+^, suggesting dependence on cathepsin B for both stimuli. We further assessed the effects of Cr_2_O_3_ particles and Cr^3+^ on galectin puncta formation – an indicator of lysosomal destabilization that can lead to cytosolic release of cathepsin B. The particles, but not the ions, induced galectin puncta formation. Together, these results suggest that 60-nm spherical Cr_2_O_3_ particles can induce lysosomal destabilization, leading to cathepsin B leakage and subsequent NLRP3 inflammasome activation. In contrast, cathepsin B-dependent IL-1β release induced by Cr^3+^ occurred without detectable lysosomal destabilization, possibly due to spatially and temporally restricted lysosomal release of limited quantities of cathepsin B into the cytosol [34,61]. Interestingly, Cr^3+^ elicited a stronger cathepsin B-dependent IL-1β release than the Cr_2_O_3_ particles, despite the absence of overt lysosomal destabilization, suggesting the existence of complementary mechanisms. The ROS-dependence of Cr^3+^ -induced IL-1β release further suggests that ROS production and other synergistic pathways may potentiate cathepsin B-mediated inflammasome activation. By contrast, lysosomal rupture may lack these synergistic signals or could even activate negative regulatory pathways that limit IL-1β release despite the release of cathepsin B [62]. A controlled release of cathepsin B could occur in proximity to inflammasome assembly sites, such as mitochondria-associated endoplasmic reticulum (ER) membranes, thereby enhancing the efficiency of inflammasome activation [34]. In contrast, lysosomal rupture might result in the diffuse or mislocalized release of cathepsins, reducing their effective concentration at critical signalling hubs.

Exposure of primary human monocytes and THP-1 (human) macrophages to spherical or irregularly-shaped 6–7-μm CoCrMo particles [24,25], as well as to Co^2+^, has been reported to activate the NLRP3 inflammasome and trigger the subsequent release of IL-1β [25]. More specifically, Caicedo *et al*. [24] reported that increasing size (up to 6 μm diameter) and shape irregularity of CoCrMo particles resulted in greater lysosomal damage and enhanced NLRP3 inflammasome activation. However, the present study found that both 3.4-μm spherical CoCrMo particles and Co^2+^ induced IL-1β release independently of the NLRP3 inflammasome. As a result, the potential of CoCrMo particles or Co^2+^ to cause lysosomal damage was not investigated. The apparent inconsistency between studies may be attributed to differences in cell type (human peripheral blood monocytes vs. murine BMDM), particle characteristics (for CoCrMo particles only, including, size and shape), and/or experimental design – for example, the use of small interfering RNA (siRNA) for *Nlrp3* knockdown compared to genetic knockout in the present study. Moreover, exposing NLRP3-deficient BMDM to CoCrMo particles increased IL-1β release, suggesting the activation of compensatory pathways in the absence of NLRP3.

Experiments using BMDM from *Casp1*^*-/-*^ mice showed that IL-1β release induced by the 3.4-μm CoCrMo particles was caspase-1 independent, whereas that induced by Co^2+^ was partially caspase-1 dependent. Interestingly, NLRP3-independent but caspase-1-dependent IL-1β release has also been reported in 3T3-L1 adipocytes exposed to TNF-α [63], as well as in RAW 264.7 macrophages stimulated with monosodium urate crystals [64]. Since exposure to Co^2+^ induced TNF-α release by BMDM (S3 Fig), the NLRP3-independent but partially caspase-1-dependent IL-1β release observed in response to Co^2+^ could involve TNF-α signaling. Although the mechanisms by which Co^2+^ induce the cleavage of inactive pro-caspase-1 into its mature form remain largely unknown, potential NLRP3-independent mechanisms have been identified. For example, in RAW 264.7 macrophages exposed to monosodium urate crystal, epidermal growth factor receptor pathway substrate 8 (Eps8) has been reported to form a complex with pro-caspase-1 and be required for its activation – suggesting that the Eps8-pro-caspase-1 complex facilitates cleavage [64].

Our observation that IL-1β release induced by 3.4-μm spherical CoCrMo particles and Co^2+^ occurred independently of NLRP3 and was either fully (CoCrMo particles) or partially (Co^2+^) caspase-1 independent, led us to investigate the potential involvement of caspase-8 in this release. Caspase-8 non-apoptotic functions have been shown to include the cleavage of IL-1β into its mature active form, either directly [37,65] or via activation of the NLRP3 inflammasome [65,66]. Furthermore, elevated levels of caspase-8 have been found in the interface membranes of aseptically loosened total hip implants, but not in control tissues (e.g., from hip dysplasia or mechanical loosening) [67–69]. In the present study, we showed that a caspase-8 inhibitor (Z-IETD-FMK) prevented IL-1β release induced by CoCrMo particles and Co^2+^, suggesting that these processes are fully caspase-8-dependent. This is particularly interesting because Co^2+^-induced IL-1β release is also partially dependent on caspase-1, even though it is independent of the NLRP3 inflammasome. We propose that Co^2+^ may be inducing IL-1β release by causing transient caspase-1 dimerization, a mechanism described by Boucher *et al*. [70]. Additionally, the enhanced release of IL-1β observed in NLPR3-deficient BMDM exposed to the CoCrMo particles or Co^2+^ may be attributed to increased caspase-8 synthesis or activity when NLRP3 is absent. The observed dependence on caspase-8 might be explained by the potential of Co^2+^ to induce the proapoptotic Bax/Bak proteins [67], which can lead to caspase-8 activation [71]. It should be noted that our findings contrast with those of Petit *et al*. [67], who observed that Cr^3+^, but not Co^2+^, increased caspase-8 expression and activity in U937 human macrophages. However, an important caveat to their study is that, unlike in non-neoplastic macrophages such as BMDM, mature caspase-8 was constitutively expressed in the U937 cells used [67]. Finally, it should be noted that caspase-8, which is capable of activating the NLRP3 inflammasome [66,72–74], may also have contributed to the NLRP3-dependent IL-1β release induced by the Cr_2_O_3_ particles or Cr^3+^.

### Limitations of the study

BMDM used in the present study were generated exclusively from female mice due to housing considerations. Although sex-based differences are not expected to alter the core immune mechanisms under investigation, they may influence the magnitude of the response. Murine BMDM are a widely used model for studying inflammatory mechanisms *in vitro*; however, species-specific differences between murine and human macrophages should be considered when extrapolating these findings to humans. Most notably, unlike in murine monocytes, human monocytes do not always require a two-step process for NLRP3 inflammasome activation. TLR4 stimulation alone has been shown to be sufficient to induce the release of IL-1β [75,76]. In the present *in vitro* study, BMDM were primed with LPS, a commonly used priming agent in NLRP3 inflammasome studies. *In vivo*, however, priming may be mediated by a variety of endogenous factors (such as DAMPs or alarmins) [19] or exogenous PAMPs (like LPS) that originate from a subclinical bacterial biofilm on the implant material surface [77,78].

The commercial availability of CoCrMo nanoparticles is limited by challenges in consistent generation, instability due to spontaneous oxidation, and the occupational hazards presented by their inherent biological toxicity. As a result, the CoCrMo particles used in the present study were larger than those typically isolated from periprosthetic tissues [11]. It should also be noted that, although both spherical and elongated CoCrMo particles are found in periprosthetic tissues, spherical particles are generally less abundant [11]. Although particle size and shape can be important factors influencing IL-1β release and lysosomal destabilization [24], particle composition is also a critical determinant. For example, our results demonstrate that Cr_2_O_3_ particles and Cr^3+^ activated similar inflammatory mechanisms, as did CoCrMo particles and Co^2+^. Given that CoCrMo alloys can release Co^2+^ through degradation, the similarity in responses induced by CoCrMo particles and Co^2+^ may reflect ion release as a contributing factor. In contrast, Cr_2_O_3_ is considered relatively chemically inert and is therefore unlikely to generate Cr^3+^ under physiological conditions. The similarity of the mechanisms activated by Cr_2_O_3_ particles and Cr^3+^ may thus be attributed to their shared elemental composition (Cr) and size congruity. Our results also demonstrated that IL-1β release by BMDM, from *Casp1*^*-/-*^ mice, exposed to CoCrMo particles was caspase-1 independent, whereas IL-1β release in response to Co^2+^ was partially dependent on caspase-1. This divergence suggests that the effects observed with CoCrMo particles were, at least in part, attributable to the particles *per se* rather than to potential release of Co^2+^.

Finally, it should be noted that although the inhibitors used in the present study are generally considered specific for their intended targets, the possibility of off-target effects cannot be fully excluded.

## Conclusion

**Fig 7:**
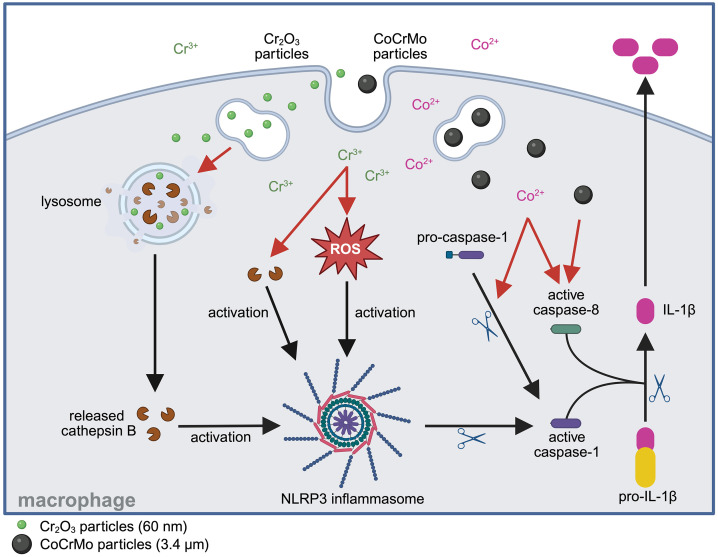
Schematic summary of the mechanisms involved in the release of IL-1β by BMDM characterized in the present study. IL-1β: interleukin-1β; BMDM: bone marrow-derived macrophages. Created in BioRender. Abdoulkader, N. (2025) https://BioRender.com/s84o209.

This study provides novel insight into the inflammatory response of macrophages triggered by CoCrMo implant wear and corrosion products. It highlights the specificity and diversity of the underlying mechanisms, revealing distinct pathways through which CoCrMo and Cr_2_O_3_ particles, as well as Co^2+^ and Cr^3+^, can induce IL-1β release in macrophages (Fig. 7). Among these pathways, NLRP3, caspase-1, and caspase-8 emerge as key mediators, positioning them as potential molecular targets for future therapeutic strategies. The activation of stimulus-specific signaling cascades underscores the complexities of implant-related inflammation while also presenting opportunities for more precise tailored therapeutic approaches. Although IL-1β represents only one component of a much broader multifaceted response, it plays a central role in periprosthetic inflammation. By elucidating the mechanisms through which wear particles and metal ions promote IL-1β-mediated inflammatory response in macrophages, this research provides a critical foundation for the development of targeted strategies to reduce inflammation and ultimately extend the functional lifespan of CoCrMo-based implants.

## Supporting information

S1 Materials and methods Purity of bone marrow-derived macrophage preparations. (PDF)

S2 Fig Characterization of BMDM cell preparation purity. (PDF)

S3 Fig IL-1β (A) and TNF-α (B) release by BMDM primed with various concentrations of LPS, then exposed to Co^2+^. (PDF)

S4 Fig Effects of Co^2+^ and Cr^3+^ on the quantification of IL-1β by ELISA. (PDF)

S5 Table Cohen’s *d* values and confidence intervals (CI). (PDF)

S6 Fig IL-1β release by BMDM, from wild-type (*wt*) and *Nlrp3*^*-/-*^ mice, exposed to nigericin. (PDF)

S7 Fig Raw immunoblot images. (PDF)

S8 Fig Trypan blue staining of BMDM detached after exposure to CoCrMo particles (A) or Cr_2_O_3_ particles (B). (PDF)
