# Supplementary material for: NLRP3 inflammasome-dependent and -independent interleukin-1β release by macrophages exposed to wear and corrosion products from CoCrMo implants
Source: PLoS One. 2025 Nov 18;20(11):e0334912. doi: 10.1371/journal.pone.0334912 (PMC12626288; doi:10.1371/journal.pone.0334912)
Supplement: S1 Materials and methods — (PDF) [file pone.0334912.s001.pdf]

Purity of bone marrow-derived macrophage preparations

Purity of the BMDM preparations was assessed by flow cytometric phenotyping using epidermal growth factor (EGF)-like module-containing mucin-like hormone receptor-like 1 (F4/80) and macrophage antigen-1 beta chain (CD11b) as surface markers of macrophages [1]. Briefly, cells from BMDM preparations were seeded in tissue culture-treated polystyrene Petri dishes (Greiner Bio-One) at a cell density of 1.6×10^5 cells/cm^2 and incubated overnight in CGM supplemented with M-CSF (5 ng/mL) to recover from harvesting. At the end of the incubation, the cells were detached using a cell detachment solution (Accutase; Innovative Cell Technologies, San Diego, CA), as per the manufacturer's instructions (15-min incubation). The detached cells were collected by centrifugation ($300 \times g$ for 5 min) then resuspended at concentration of 1.0×10^6 cells/mL in staining buffer composed of DPBS without Ca^{2+} and Mg^{2+}, supplemented with 2% (v/v) heat-inactivated FBS and 2 mM EDTA (Fisher Scientific). Aliquots (50 µL) of cell suspension, at a nominal concentration of 2.0×10^6 cells/mL, were incubated 10 min on ice with an anti-mouse CD16 (Fc gamma III Receptor)/CD32 (Fc gamma II Receptor) antibody (0.5×10^{-2} µg/µL final; clone 93; ThermoFisher Scientific) to block the Fc receptors. The cells were then incubated on ice for an additional 20 min with allophycocyanin (APC)-labeled anti-mouse F4/80 antibody ($5 \times 10^{-}3$ µg/µL final; clone QA17A29; BioLegend, San Diego, CA), fluorescein isothiocyanate (FITC)-labeled anti-mouse/human CD11b (1.25×10^{-3} µg/µL final; clone M1/70; BioLegend), and 7-aminoactinomycin D (7-AAD; 3 µg/mL final; StemCell Technologies, Vancouver, BC). At the end of the incubation, the cells were collected by centrifugation ($300 \times g$ for 5 min), washed once with ice-cold staining buffer, and resuspended in 200 µL of ice-cold staining buffer. The cell suspension was then transferred into 5-mL round-bottom polystyrene culture tubes (Greiner Bio-One). Cells were analyzed immediately by flow cytometry using an LSRFortessa Cell Analyzer

(BD Biosciences, Franklin Lakes, NJ) and data were analyzed using FlowJo software v10.10 (BD Biosciences).

Reference

1. Liu L, Stokes JV, Tan W, Pruett SB. An optimized flow cytometry panel for classifying macrophage polarization. J Immunol Methods. 2022;511:113378.
