# Supplementary material for: NLRP3 inflammasome-dependent and -independent interleukin-1β release by macrophages exposed to wear and corrosion products from CoCrMo implants
Source: PLoS One. 2025 Nov 18;20(11):e0334912. doi: 10.1371/journal.pone.0334912 (PMC12626288; doi:10.1371/journal.pone.0334912)
Supplement: S2 Fig — (PDF) [file pone.0334912.s002.pdf]

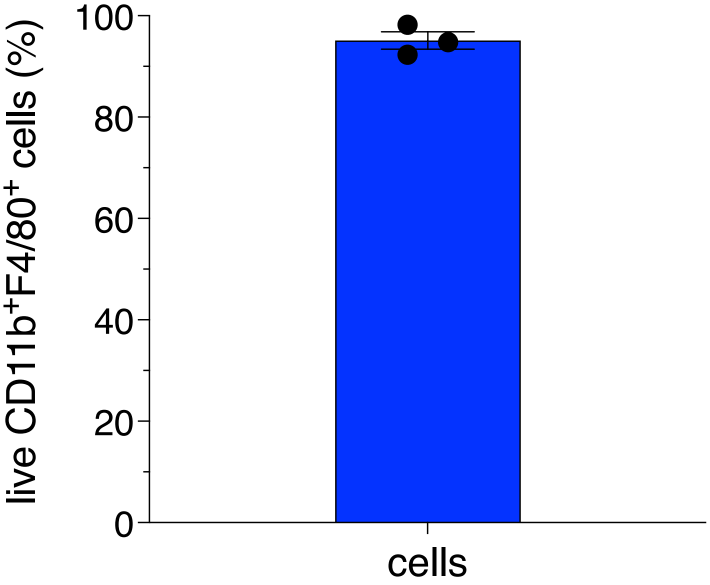

S2 Fig. Characterization of BMDM cell preparation purity. Bone marrow-derive macrophages (BMDM) cell preparations from wild-type (*wt*) mice were stained with an allophycocyanin (APC)-labelled anti-mouse F4/80 antibody, a fluorescein isothiocyanate (FITC)-labelled anti-mouse CD11b antibody, and the 7-aminoactinomycin D (7-AAD) viability dye, then analyzed by flow cytometry. Data are presented as mean ± SEM of 3 independent experiments.
