# Supplementary material for: NLRP3 inflammasome-dependent and -independent interleukin-1β release by macrophages exposed to wear and corrosion products from CoCrMo implants
Source: PLoS One. 2025 Nov 18;20(11):e0334912. doi: 10.1371/journal.pone.0334912 (PMC12626288; doi:10.1371/journal.pone.0334912)
Supplement: S3 Fig — (PDF) [file pone.0334912.s003.pdf]

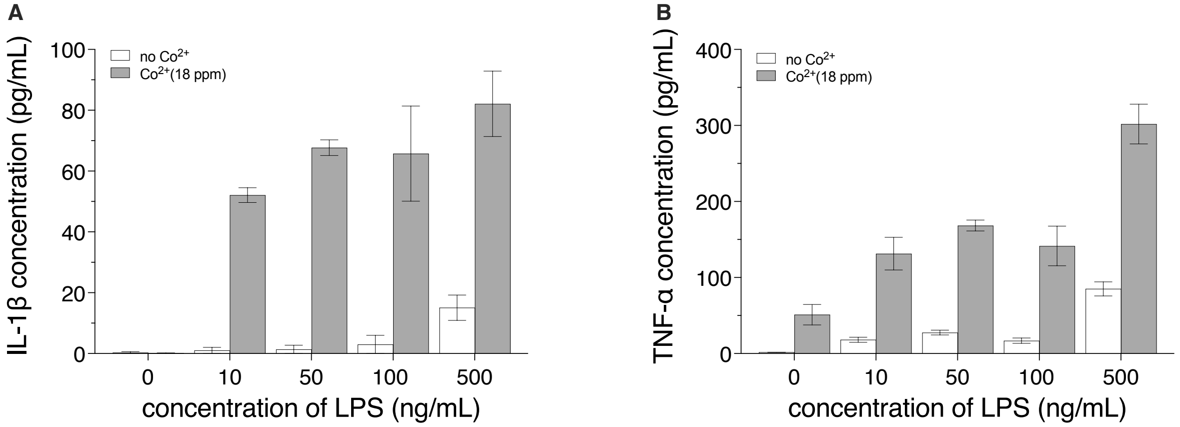


**S3 Fig. IL-1β (A) and TNF-α (B) release by BMDM primed with various concentrations of LPS then exposed to $Co^{2+}$**. Bone marrow-derived macrophages (BMDM) from wild-type (*wt*) mice were primed with lipopolysaccharide (LPS; 0, 10, 50, 100, or 500 ng/mL) for 3 h, then exposed to $Co^{2+}$ (0, 18 ppm) for 18 h. Interleukin IL-1β (IL-1β) and tumor necrosis factor alpha (TNF-α) release were quantified by enzyme-linked immunosorbent assay (ELISA). Data are presented as mean ± SEM of 2 independent experiments.
