# Supplementary material for: NLRP3 inflammasome-dependent and -independent interleukin-1β release by macrophages exposed to wear and corrosion products from CoCrMo implants
Source: PLoS One. 2025 Nov 18;20(11):e0334912. doi: 10.1371/journal.pone.0334912 (PMC12626288; doi:10.1371/journal.pone.0334912)
Supplement: S4 Fig — (PDF) [file pone.0334912.s004.pdf]

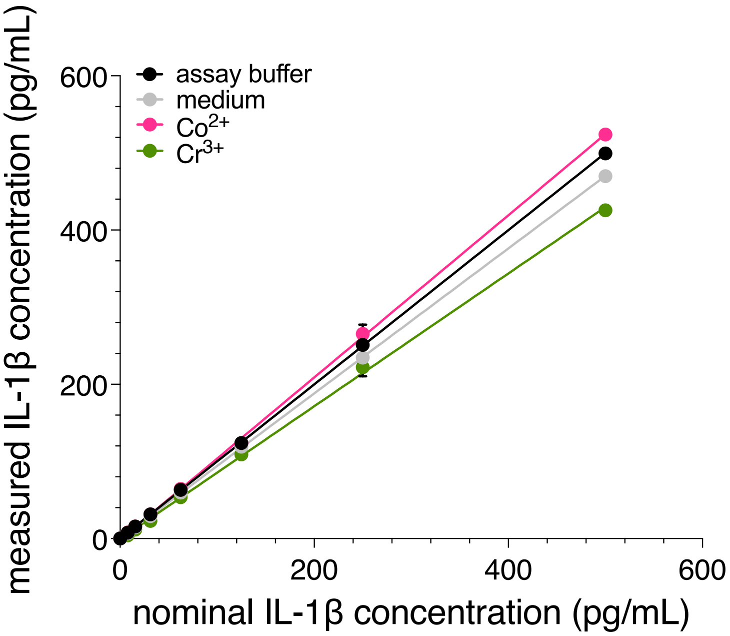

S4 Fig. Effects of Co^{2+} and Cr^{3+} on the quantification of IL-1β by ELISA. A standard solution of interleukin-1β (IL-1β) was spiked with medium (baseline), Co^{2+} (4.5 ppm final), or Cr^{3+} (75 ppm final). These concentrations of Co^{2+} and Cr^{3+} correspond to the highest concentrations present in the diluted experimental supernatants used for IL-1β quantification by enzyme-linked immunosorbent assay (ELISA). Linear regression analysis for assay buffer: slope = 0.99 ± 0.009, $R^2 = 0.99$, $F(1, 14) = 54470$; medium: slope = 0.94 ± 0.004, $R^2 = 0.99$, $F(1, 14) = 181231$, $p < 0.001$; Co^{2+}: slope = 1.05 ± 0.02, $R^2 = 0.99$, $F(1, 14) = 20839$, $p < 0.001$); and Cr^{3+}: slope = 0.86 ± 0.02, $R^2 = 0.99$, $F(1, 14) = 11345$, $p < 0.001$). Data are presented as mean ± SEM of two technical replicates. SEM ≤ 10 are covered by the symbols. Medium refers to complete growth medium (see Materials and methods).
