# Supplementary material for: NLRP3 inflammasome-dependent and -independent interleukin-1β release by macrophages exposed to wear and corrosion products from CoCrMo implants
Source: PLoS One. 2025 Nov 18;20(11):e0334912. doi: 10.1371/journal.pone.0334912 (PMC12626288; doi:10.1371/journal.pone.0334912)
Supplement: S5 Table — (PDF) [file pone.0334912.s005.pdf]

FIGURE 1†		
**panel A**		
**comparison**	**Cohen's *d***	**confidence interval**
wt 0 vs *wt* 1.7	3.3	[0.9, 5.8]
wt 0 vs *wt* 2.4	3.3	[0.9, 5.8]
wt 1.7 vs *Nlrp3*$^{-/-}$ 1.7	-5.4	[-9.0, -2.0]
wt 2.4 vs *Nlrp3*$^{-/-}$ 2.4	-4.2	[-7.0, -1.3]
**panel B**		
**comparison**	**Cohen's *d***	**confidence interval**
wt 0 vs *wt* 300	5.4	[2.0, 8.9]
wt 300 vs *Nlrp3*$^{-/-}$ 300	-5.7	[-9.2, -2.1]
**panel C**		
**comparison**	**Cohen's *d***	**confidence interval**
wt 0 vs *wt* 1.7	5.0	[2.2, 7.8]
wt 1.7 vs *Casp1*$^{-/-}$ 1.7	-4.9	[-7.7, -2.1]
**panel D**		
**comparison**	**Cohen's *d***	**confidence interval**
wt 0 vs *wt* 300	3.9	[1.4, 6.5]
wt 300 vs *Casp1*$^{-/-}$ 300	-3.2	[-5.6, -0.8]

† Abbreviations are expanded in the main document.

**FIGURE 2†**		
**panel A**		
**comparison**	**Cohen's *d***	**confidence interval**
no NAC 0 vs no NAC 1.7	-0.4	[-2.0, 1.2]
no NAC 0 vs no NAC 2.4	0.1	[-1.7, 1.5]
**panel B**		
**comparison**	**Cohen's *d***	**confidence interval**
no NAC 0 vs no NAC 300	4.7	[1.7, 7.9]
no NAC 300 vs NAC 300	-4.4	[-7.4, -1.5]

† Abbreviations are expanded in the main document.

FIGURE 3†		
**panel A**		
**comparison**	**Cohen's *d***	**confidence interval**
no CA-074Me 0 vs no CA-074Me 2.5	1.8	[-0.1, 3.7]
no CA-074Me 2.5 vs CA-074Me 2.5	-2.2	[-4.3, -0.2]
**panel B**		
**comparison**	**Cohen's *d***	**confidence interval**
no CA-074Me 0 vs no CA-074Me 300	4.5	[1.5, 7.5]
no CA-074Me 300 vs CA-074Me 300	-4.3	[-7.2, -1.4]

† Abbreviations are expanded in the main document.

FIGURE 4†		
**panel A**		
**comparison**	**Cohen's *d***	**confidence interval**
wt 0 vs *wt* 35	3.7	[1.1, 6.3]
wt 0 vs *wt* 70	4.0	[1.2, 6.7]
wt 35 vs *Nlrp3*$^{-/-}$ 35	2.0	[0.0, 3.9]
wt 70 vs *Nlrp3*$^{-/-}$ 70	2.9	[0.6, 5.2]
**panel B**		
**comparison**	**Cohen's *d***	**confidence interval**
wt 0 vs *wt* 18	3.1	[0.7, 5.5]
wt 18 vs *Nlrp3*$^{-/-}$ 18	3.3	[0.8, 5.7]

† Abbreviations are expanded in the main document.

FIGURE 5†		
**panel A**		
**comparison**	**Cohen's *d***	**confidence interval**
wt 0 vs *wt* 70	4.2	[1.3, 7.0]
**panel B**		
**comparison**	**Cohen's *d***	**confidence interval**
wt 0 vs *wt* 18	9.3	[4.5, 14]
wt 18 vs *Casp1*$^{-/-}$ 18	-3.6	[-6.0, -1.2]

† Abbreviations are expanded in the main document.

**FIGURE 6†**		
**panel A**		
**comparison**	**Cohen's *d***	**confidence interval**
no Z-IETD-FMK 0 vs no Z-IETD-FMK 100	2.7	[0.5, 4.9]
no Z-IETD-FMK 100 vs Z-IETD-FMK 100	-2.7	[-4.9, -0.5]
**panel B**		
**comparison**	**Cohen's *d***	**confidence interval**
no Z-IETD-FMK 0 vs no Z-IETD-FMK 18	3.1	[0.7, 5.5]
no Z-IETD-FMK 18 vs Z-IETD-FMK 18	-3.0	[-5.4, -0.7]

† Abbreviations are expanded in the main document.
