# Supplementary material for: NLRP3 inflammasome-dependent and -independent interleukin-1β release by macrophages exposed to wear and corrosion products from CoCrMo implants
Source: PLoS One. 2025 Nov 18;20(11):e0334912. doi: 10.1371/journal.pone.0334912 (PMC12626288; doi:10.1371/journal.pone.0334912)
Supplement: S6 Fig — (PDF) [file pone.0334912.s006.pdf]

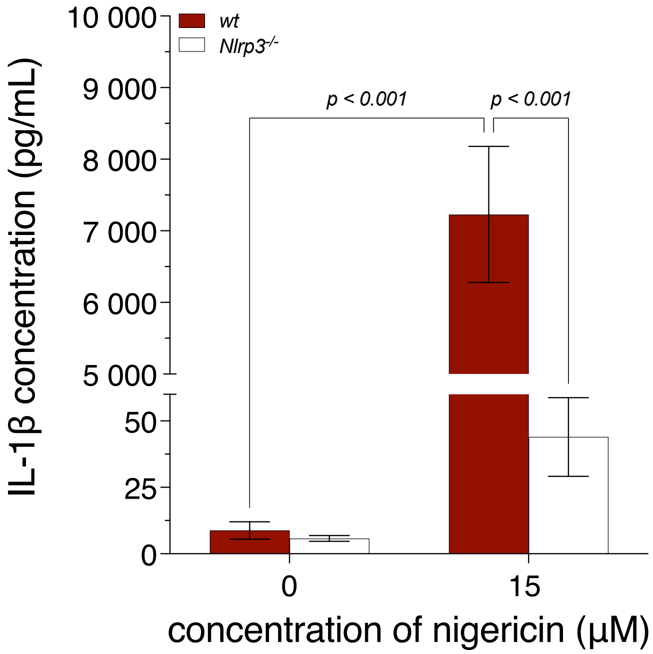

S6 Fig. IL-1β release by BMDM, from wild-type (*wt*) and *Nlrp3*$^{-/-}$ mice, exposed to nigericin. Bone marrow-derived macrophages (BMDM) were primed with 500 ng/mL of lipopolysaccharide for 3 h, then exposed to nigericin (0, 15 µM) for 18 h. Interleukin-1β (IL-1β) release was quantified by enzyme-linked immunosorbent assay (ELISA). A two-way analysis of variance (ANOVA) was performed. Since an interaction ($p < 0.001$) was detected, the Holm-Šídák multiple-comparison post hoc test was performed. Data are presented as mean ± SEM of 3 independent experiments.
