# Supplementary material for: NLRP3 inflammasome-dependent and -independent interleukin-1β release by macrophages exposed to wear and corrosion products from CoCrMo implants
Source: PLoS One. 2025 Nov 18;20(11):e0334912. doi: 10.1371/journal.pone.0334912 (PMC12626288; doi:10.1371/journal.pone.0334912)
Supplement: S7 Fig — (PDF) [file pone.0334912.s007.pdf]

western blot no. 1

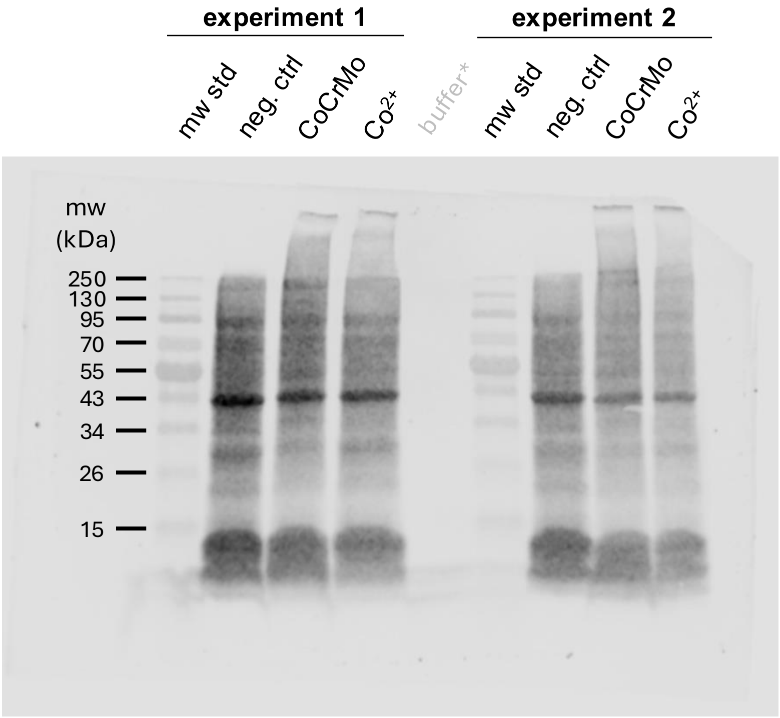

*Loading buffer (1X)

total protein stain

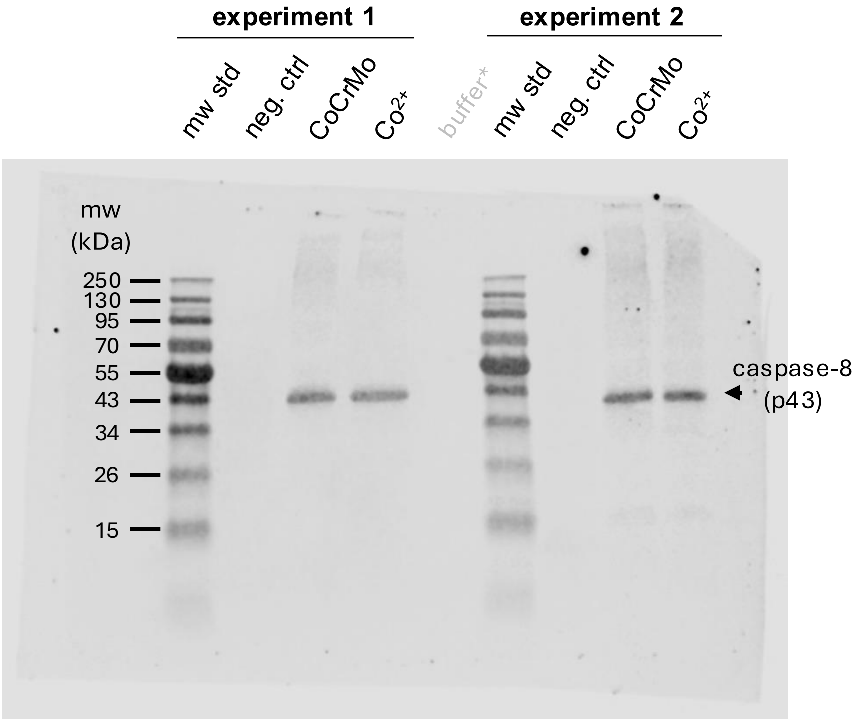

*Loading buffer (1X)

caspase-8 immunoblot

western blot no. 2

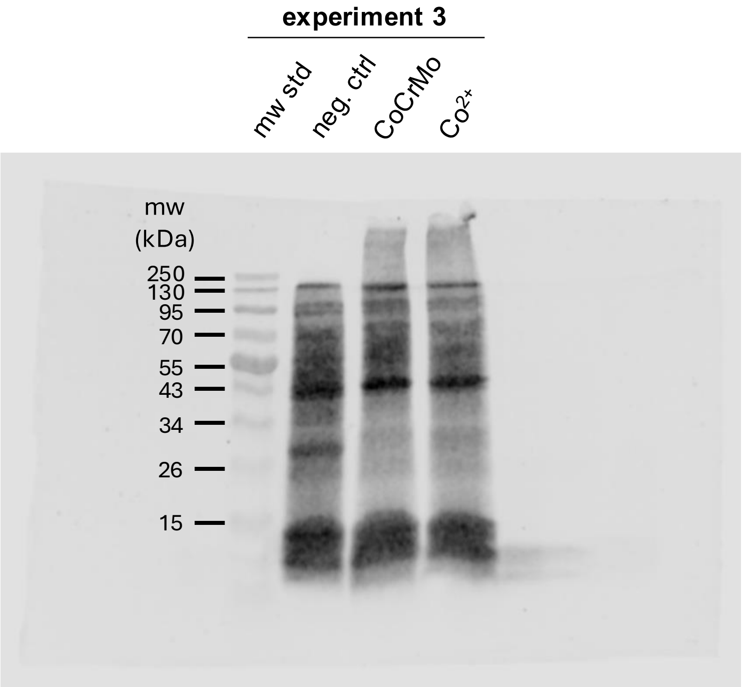

*Loading buffer (1X)

total protein stain

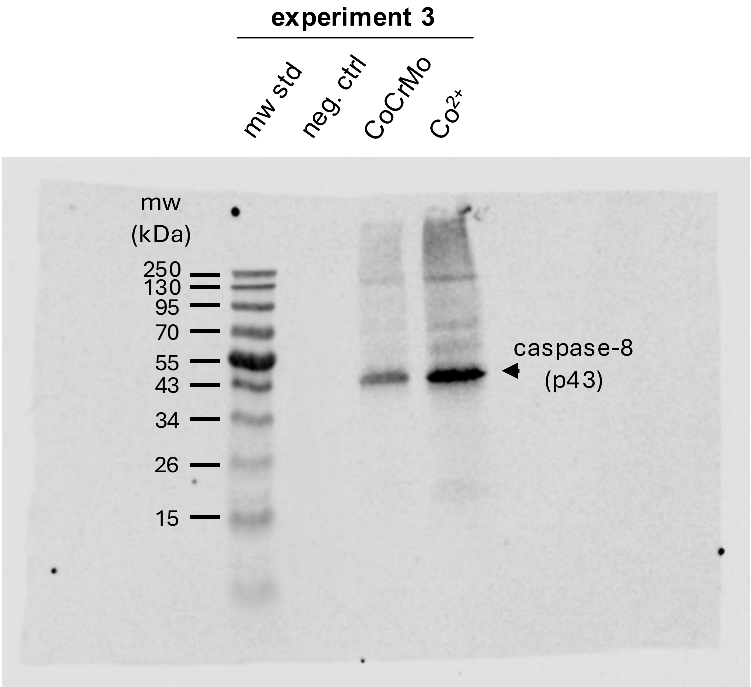

*Loading buffer (1X)

caspase-8 immunoblot
