# Supplementary material for: NLRP3 inflammasome-dependent and -independent interleukin-1β release by macrophages exposed to wear and corrosion products from CoCrMo implants
Source: PLoS One. 2025 Nov 18;20(11):e0334912. doi: 10.1371/journal.pone.0334912 (PMC12626288; doi:10.1371/journal.pone.0334912)
Supplement: S8 Fig — (PDF) [file pone.0334912.s008.pdf]

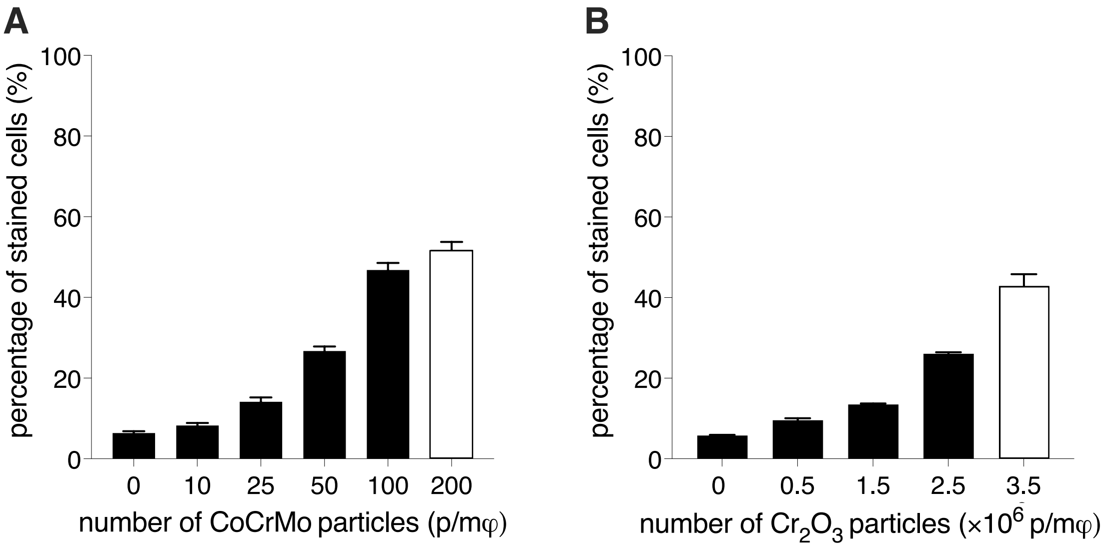

S8 Fig. Trypan blue staining of BMDM detached after exposure to CoCrMo particles (A) or Cr_2O_3 particles (B). Bone marrow-derived macrophages (BMDM) were primed with 500 ng/mL of lipopolysaccharide for 3 h, then exposed to the indicated concentration of CoCrMo or Cr_2O_3 particles for 18 h. Adherent cells were washed with ice-cold Dulbecco's phosphate buffered saline without Ca^{2+} and Mg^{2+}, incubated 15 min at room temperature in an enzymatic cell detachment solution (Accutase), and detached by gentle pipetting. Cells were resuspended in complete growth medium and counted by dye-exclusion hemocytometry using trypan blue (0.04% w/v, final concentration). Data are presented as means ± SEM from a single experiment performed in triplicate (**A**) or duplicate (**B**). Bars in white represent concentrations that extend beyond the experimental range used in the present study and are included for comparative purposes. p/mφ: particles per macrophage.

Note on date interpretation: The effects of particles on cell staining are likely overestimated, as particle-engorged cells are particularly susceptible to mechanical damage during detachment.
